# A Rational Diagnostic Algorithm for the Identification of *ALK* Rearrangement in Lung Cancer: A Comprehensive Study of Surgically Treated Japanese Patients

**DOI:** 10.1371/journal.pone.0069794

**Published:** 2013-08-01

**Authors:** Kazuya Takamochi, Kengo Takeuchi, Takuo Hayashi, Shiaki Oh, Kenji Suzuki

**Affiliations:** 1 Department of General Thoracic Surgery, Juntendo University School of Medicine, Tokyo, Japan; 2 Pathology Project for Molecular Targets, The Cancer Institute, Japanese Foundation for Cancer Research, Tokyo, Japan; 3 Department of Human Pathology, Juntendo University School of Medicine, Tokyo, Japan; The Chinese University of Hong Kong, Hong Kong

## Abstract

**Background:**

*EML4-ALK* fusion gene is found in only a small subset (2–6%) of non-small cell lung cancer. There is an urgent need to establish a rational diagnostic algorithm to identify this rare but important fusion in lung cancer.

**Methods:**

We performed a comprehensive analysis of *EGFR/KRAS* mutation and *ALK* rearrangement in a total of 360 surgically resected lung cancers. *ALK* rearrangement was examined by 3 analyses: multiplex reverse transcription-PCR, fluorescent *in situ* hybridization (FISH), and immunohistochemistry (IHC) with the intercalated antibody-enhanced polymer method. A scoring system was used for IHC (iScore). A test set (202 patients with unselected lung cancer) was used for proposing a diagnostic algorithm. This diagnostic algorithm was validated in 158 patients with *EGFR* and *KRAS* mutation-negative adenocarcinoma.

**Results:**

*ALK* rearrangement was identified in 2 patients (1.0%) from the test set and both adenocarcinomas were negative for *EGFR* and *KRAS* mutations. The results of FISH and RT-PCR were completely matched. The highest iScore 3 was found only in the 2 positive cases. A diagnostic algorithm was proposed: IHC screening for *ALK* rearrangement followed by confirmatory FISH. In the validation set, 8 cases (5.1%) had iScore 3 and were positive for FISH, while the other cases had iScore 0 and were negative for FISH.

**Conclusions:**

Screening for *ALK* rearrangement by IHC followed by confirmatory FISH is a rational diagnostic algorithm. If needed, patients may be selected for screening *ALK* rearrangement by their *EGFR* and *KRAS* mutation status.

## Introduction

Significant advances in the molecular targeted therapy for non-small cell lung cancer (NSCLC) have been made over the past 10 years. In 2004, the identification of somatic mutations in the *epidermal growth factor receptor* (*EGFR*) gene provided the first glimpse of a clinically relevant NSCLC oncogene [1], [2]. Currently, EGFR tyrosine kinase inhibitors (TKIs), such as gefitinib and erlotinib, are the first-line treatments for patients with advanced *EGFR* mutated NSCLC. In 2007, the first fusion oncogene, the *echinoderm microtubule- associated protein-like 4* (*EML4*)-*anaplastic lymphoma kinase* (*ALK*) gene, was identified in NSCLC [3]. *EML4*-*ALK* is an oncogenic driver and activates downstream signaling pathways. A recent phase I trial showed a dramatic response to the ALK inhibitor (crizotinib) in *EML4*-*ALK*-positive NSCLCs: the overall response rate was 57% and the disease control rate was 90% [4]. This led to accelerated approval of crizotinib by the Food and Drug Administration for treatment of advanced NSCLC with *ALK* rearrangements. Phase 3 clinical trials are under way in which clinical outcomes of crizotinib-treated patients are compared to those receiving standard first- and second-line therapies in advanced *ALK*-positive NSCLCs.

*EGFR* mutation is a relatively frequent driver mutation in lung adenocarcinoma and is found in approximately 10% of white patients and in over 40% of East Asian patients [5]. However, *ALK* rearrangements have been found in only a small subset of NSCLC (2–5%) and lung adenocarcinoma (4–6%) cases, regardless of race [6]–[14]. Several methods are currently being used to identify *ALK* rearrangements: reverse transcription PCR (RT-PCR), fluorescent *in situ* hybridization (FISH), and immunohistochemistry (IHC). These methods have different advantages and disadvantages and it remains to be determined which is the best method for large-scale screening of *ALK* rearrangement in lung cancer in a clinical setting.

Therefore, there is an urgent need to establish a rational diagnostic algorithm to identify this rare but important rearrangement in lung cancer in regular clinical practice. Here, we report on which methods and which combinations should be used for accurate diagnosis.

## Materials and Methods

### Ethics Statement

This study was conducted on specimens stored in the tissue bank, with the approval of the institutional review board (IRB) of Juntendo University School of Medicine. According to the tissue bank protocol, in order to collect specimens for studies that would be approved by the IRB in the future, we obtained written consent from patients prior to surgery for the collection and storage of specimens during surgery. The contents of this study were deemed ethically acceptable, and the IRB approved the use of the specimens stored in the tissue bank without obtaining new informed consent.

### Test Set

Between March 2010 and February 2011, 231 patients with primary lung cancer underwent pulmonary resection. Frozen and formalin-fixed paraffin-embedded (FFPE) tissue samples from 202 patients with NSCLCs (148 adenocarcinomas, 39 squamous cell carcinomas, 6 adenosquamous carcinomas, 4 large cell neuroendocrine carcinomas, 3 small cell carcinomas, and 2 pleomorphic carcinomas) were used as a test set to identify *ALK* rearrangement. All cases were examined by multiplex RT-PCR (using frozen materials), FISH, and IHC (using FFPE tissues). Interpretations of these molecular analyses for *ALK* rearrangement were performed independently without the knowledge of the results of each examination. We proposed a diagnostic algorithm for the identification of *ALK* rearrangement based on the combination of the significant pathological and molecular predictors and the useful diagnostic methods.

### Validation Set

Next, the proposed diagnostic algorithm for *ALK* rearrangement was validated using an additional 158 consecutive patients with *EGFR* and *KRAS* mutation-negative adenocarcinoma who underwent surgical resection between March 2011 and April 2012. *ALK* rearrangement analyses were performed for all samples by both FISH and IHC. Interpretations of FISH and IHC were performed independently without the knowledge of the results of each examination. *ALK* rearrangement was also evaluated by RT-PCR for *ALK* positive cases by FISH or IHC, when sufficient RNA samples extracted from tumor tissues were available.

In both sets, cases positive for *ALK* rearrangement by FISH and/or by multiplex RT-PCR were defined to be ALK-positive. Neither chemotherapy, radiotherapy, nor molecular target therapy using EGFR-TKIs or ALK inhibitor was performed preoperatively on any of the patients in this study.

### *ALK* Multiplex RT-PCR

In the operating room, 3–5 mm^3^ cubes of fresh lung cancer tissue were dissected and immediately placed in 1.0 ml of RNAlater RNA Stabilization Reagent (Qiagen, GmbH, Germany, Hilden) for 24–48 h at 4°C for RNA stabilization. Thereafter, tumor specimens were stored at −80°C until RNA extraction. Total RNA was extracted from frozen tissue sections according to the standard protocol. Multiplex RT-PCR was performed for detection of *EML4*-*ALK* fusion variants, such as variant 1, 2, 3a, and 3b [15]. If any PCR products other than these 4 variants were identified by gel electrophoresis, their sequences were examined by 3130xl Genetic Analyzer (Life Technologies, Carlsbad, CA, USA).

### *ALK* FISH

**Figure 1:**
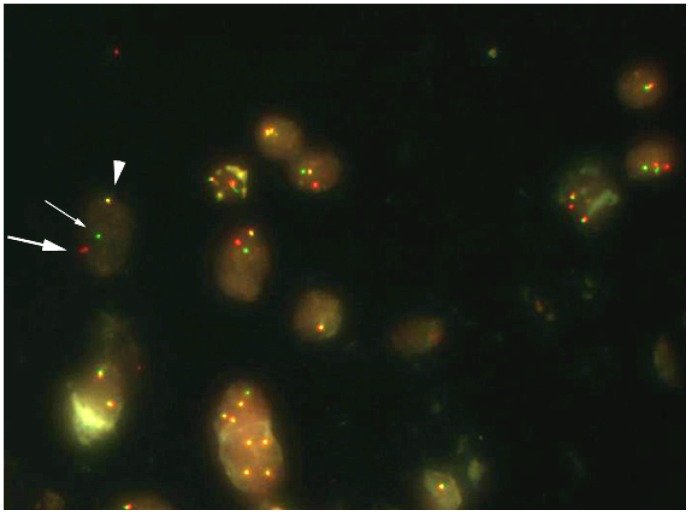
Fluorescent in situ hybridization (FISH) assay for *ALK* using dual-color break-apart probe. Distinct red (thick arrow) and green (thin arrow) break apart signals indicate the *ALK* rearrangement, and a fusion signal (arrow head) represents wild-type *ALK* gene.

FISH analysis was performed on 4 µm FFPE tissue sections using *ALK* break-apart probes [Vysis LSI ALK (2p23) Dual Color, Break Apart Rearrangement Probe; Abbott Molecular, Chicago, IL, USA], and 3*′* (red) and 5*′* (green) signals separated by ≥2 signal diameters were considered split (Figure 1). Specimens were considered positive for *ALK* rearrangement when more than 15% of tumor cells demonstrated split signals or single red signals. At least 50 tumor cells were examined per specimen.

### ALK IHC

We prepared 4 µm FFPE tissue sections for IHC analysis, and they were placed on silane-coated slides. ALK Detection Kit (Nichirei Bioscience, Tokyo, Japan), which is based on the intercalated antibody-enhanced polymer (iAEP) method [16] and includes the 5A4 clone as the anti-ALK primary antibody, was used for IHC. This highly sensitive method enables a reliable detection of various types of ALK fusion proteins in FFPE tissue samples, while it is usually difficult to detect EML4-ALK by the conventional IHC method owing to the weak activity of the *EML4* promoter that drives the expression of *EML4*-*ALK* mRNA.

Tumor cells in the cytoplasm that stained stronger than negative control cells were defined as IHC positive. Semi-quantitative assessment was done by estimating the percentage of IHC-positive tumor cells. ALK IHC scores using the iAEP method (iScore) were assigned as follows: 0 = no stained cells; 1 = 0–50% stained tumor cells; 2 = 50–80% stained tumor cells or >80% stained tumor cells with marked variability of staining intensity (“checker board pattern”); 3 = >80% stained tumor cells without marked variability of staining intensity.

### *EGFR* and *KRAS* Mutation Analyses

Genomic DNA was extracted from 3–5 mm^3^ cubes of frozen fresh lung cancer tissue samples from surgically resected specimens. The peptide nucleic acid-locked nucleic acid (PNA-LNA) PCR clamp method [17] was used to identify *EGFR* mutations. The PNA-mediated PCR clamping method [18] was used to identify *KRAS* mutations at codons 12 and 13. Molecular analyses for the *EGFR* and *KRAS* mutations and the *ALK* rearrangement were conducted at Mitsubishi Chemical Medience Corporation in Tokyo, Japan.

### Statistical Analyses

Clinicopathological factors such as age, gender, preoperative serum carcinoembryonic antigen (CEA) level, histology, pathological stage, tumor size, nodal status, lymphatic permeation, and blood vessel invasion were compared between the test set and the validation set and between the patients with *ALK*-positive and *ALK*-negative adenocarcinomas. Chi-square test or Fisher’s exact test was used for statistical analysis. A *P* -value <0.05 was considered statistically significant. All statistical analyses were performed using the SPSS statistical software package (version 20.0, SPSS Inc., Chicago, IL, USA).

## Results

Comparisons of clinicopathological and molecular characteristics of patients between the test set and the validation set are summarized in Table 1. There were more patients with pathological stage I disease in the validation set than the test set (*P* = 0.004). The proportion of patients with a tumor larger than 30 mm (*P* = 0.015) and patients with a tumor with lymphatic permeation (*P* = 0.023) or vascular invasion (*P* = 0.011) were significantly higher in the test set compared to the validation set.

**Table 1:** Comparison of clinicopathological characteristics of the patients between the test and validation set.

	Test set,n (%)	Validationset, n (%)	*P* a
Gender			0.135
Male	122 (60)	83 (53)	
Female	80 (40)	75 (47)	
Age (yr)			0.192
≤40	5 (2)	6 (4)	
>40	197 (98)	152 (96)	
Smoking			0.361
Never (pack-year ≤5)	84 (42)	70 (44)	
Smoker (pack-year >5)	118 (58)	88 (56)	
Serum CEA level			0.597
Normal	103 (51)	85 (54)	
Elevated	99 (49)	73 (46)	
Histology			<0.001 b
Adenocarcinoma	148 (73)	158 (100)	
SCC	39 (19)	0 (0)	
Others	15 (7)	0 (0)	
Pathological stage			0.004
I	116 (57)	114 (72)	
II–IV	86 (43)	44 (28)	
Tumor size (mm)			0.015
≤30	121 (60)	114 (72)	
>30	81 (40)	44 (28)	
Pathological nodal status			0.286
N0	146 (72)	122 (77)	
N1/N2	56 (28)	36 (23)	
Lymphatic permeation			0.023
Positive	93 (46)	54 (34)	
Negative	109 (54)	104 (66)	
Vascular invasion			0.011
Positive	96 (48)	54 (34)	
Negative	106 (52)	104 (66)	
*EGFR*			<0.001
Mutation (+)	66 (33)	0 (0)	
Mutation (−)	136 (67)	158 (100)	
*KRAS*			<0.001
Mutation (+)	26 (13)	0 (0)	
Mutation (−)	176 (87)	158 (100)	

a Chi-square test.

b *P* value was derived from a comparison between adenocarcinoma and non-adenocarcinoma.

CEA, carcinoembryonic antigen; SCC, squamous cell carcinoma.

### Proposal of a Rational Diagnostic Algorithm for *ALK* Rearrangement

**Figure 2:**
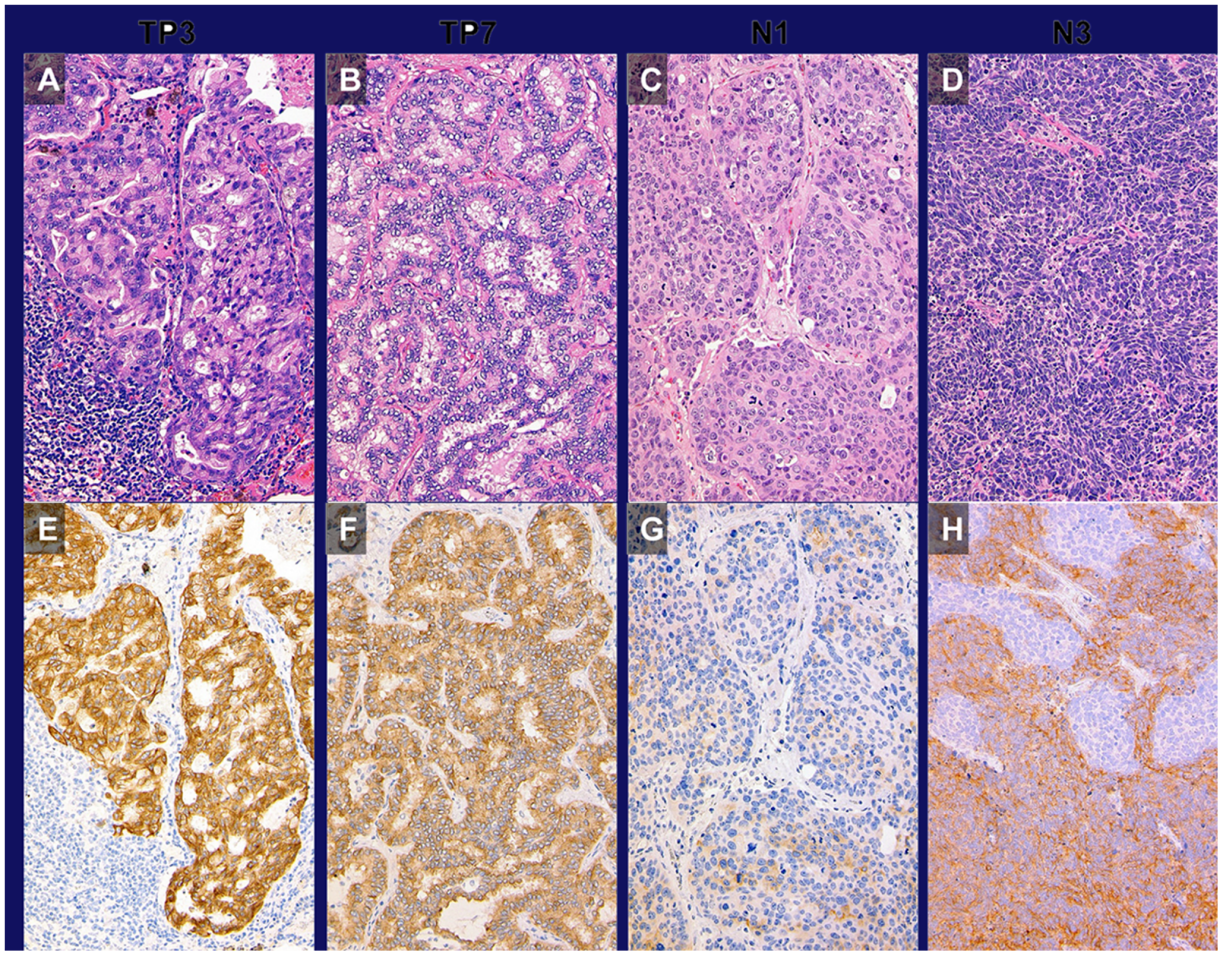
Histological features of *ALK*-positive and *ALK*-negative adenocarcinomas on H&E stain (A, B, C, D) and on ALK immunostaining by the iAEP method (E, F, G, H). P3 and P7 (A and B, respectively), adenocarcinomas with mucinous cribriform pattern, showed iScore 3 (E and F, respectively); N1 (C), squamous cell carcinoma showed iScore 1 (G); N3 (D), small cell carcinoma showed iScore 2 (H).

*ALK* rearrangement was identified in 2 samples (1.0%) from the test set and both cases were adenocarcinoma (2/148 adenocarcinomas; 1.4%). *ALK* rearrangement was consistently identified by all 3 methods. The results were completely matched between RT-PCR and FISH. iScores were 3 in the 2 ALK-positive cases, 2 in 1 negative case, and 1 in 2 negative cases (Figure 2). *ALK* rearrangements and *EGFR* and *KRAS* mutations were observed in a mutually exclusive manner as reported in many papers [6], [7], [9], [12], [13], [19]. Therefore, we considered that the diagnostic process of *ALK* rearrangement could be simplified as follows: screening by IHC with iAEP method followed by confirmatory FISH in *EGFR* and *KRAS* mutation-negative lung adenocarcinomas.

### Validation of a Proposed Diagnostic Algorithm for *ALK* Rearrangement

**Figure 3:**
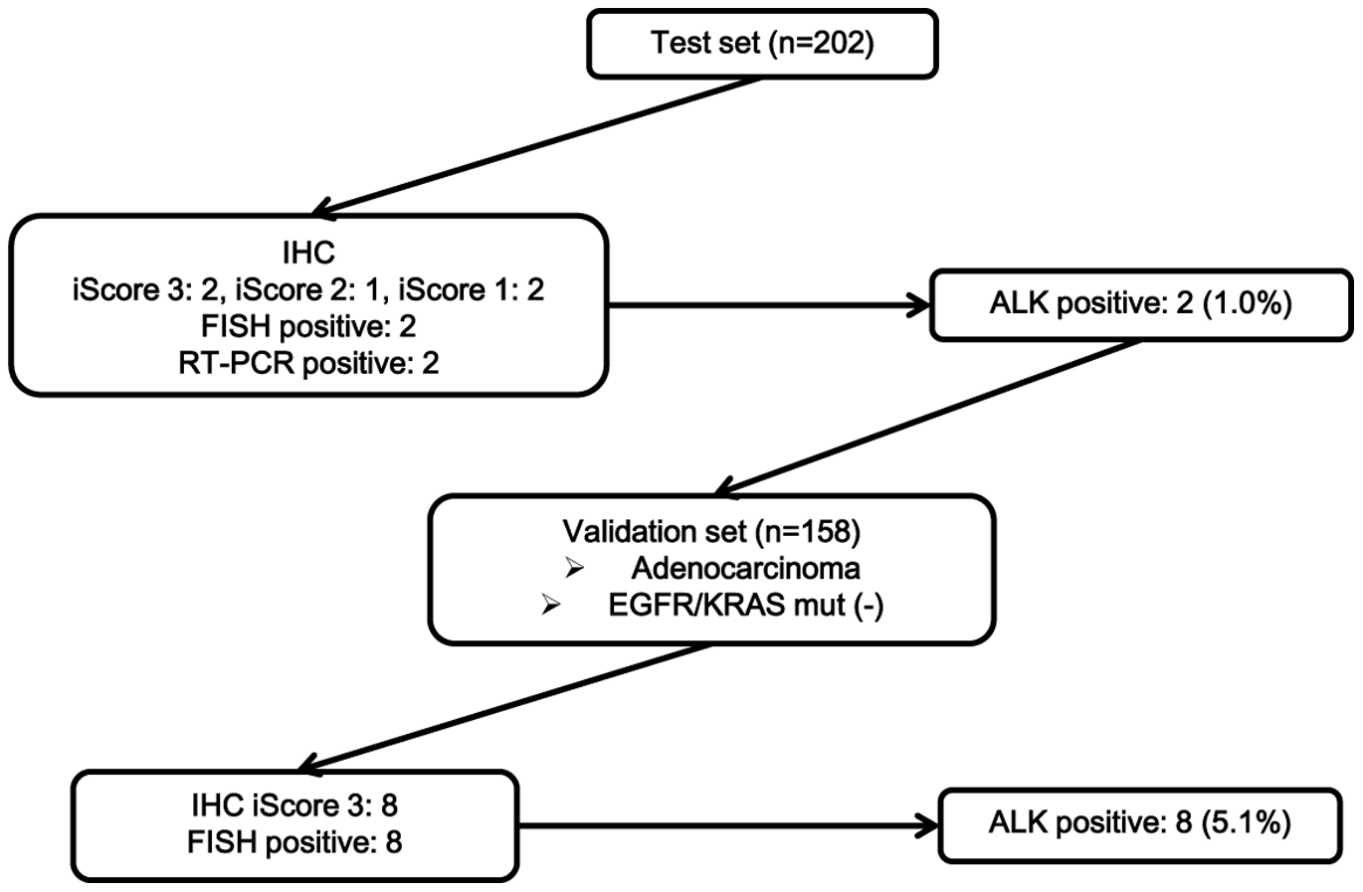
The results of assessment for *ALK* rearrangement in the test and validation set.

In 158 *EGFR* and *KRAS* mutation-negative adenocarcinomas, 8 cases (5.1%) with iScore 3 and 150 cases with iScore 0 were identified. FISH were positive in all of the 8 iScore 3 cases, and negative in the other cases. The presence of *ALK* rearrangement was evaluated by RT-PCR in 5 of the 8 true positive cases. All of the 5 cases were positive by RT-PCR. Those results were summarized in Figure 3. The correlations of IHC and FISH in all 360 patients (test and validation sets) are shown in Table 2.

**Table 2:** Correlation of the results between *ALK* IHC and FISH in all patients.

	*ALK* FISH		
ALK IHC (iScore)	Positive	Negative	Total (%)
0	0	347	347 (96.4)
1	0	2	2 (0.6)
2	0	1	1 (0.3)
3	10	0	10 (2.8)
Total (%)	10 (2.8)	350 (97.2)	360 (100)

IHC, immunohistochemistry; FISH, fluorescent *in situ* hybridization.

### The Clinicopathological Characteristics of ALK-positive Adenocarcinoma

The clinicopathological characteristics of adenocarcinoma according to *ALK* rearrangement status are summarized in Table 3. Although the size of tumor was smaller (*P* = 0.034), ALK-positive adenocarcinoma showed more aggressive biological characteristics compared to ALK-negative adenocarcinoma: more advanced stage disease (*P* = 0.014) and more frequent lymph node involvement (*P* = 0.002). *ALK* rearrangements were found only in adenocarcinoma histology; however, various predominant histological subtypes were observed among them. No specific morphological characteristic for ALK-positive adenocarcinomas was identified in our cases. Furthermore, intratumoral histological heterogeneity was also observed in each tumor (Table 4).

**Table 3:** Clinicopathological characteristics of adenocarcinoma according to *ALK* rearrangement status.

	Total, n (%)	*ALK* positive, n (%)	*ALK* negative, n (%)	*P* a
Gender				0.303
Male	117 (53)	4 (40)	113 (54)	
Female	104 (47)	6 (60)	98 (46)	
Age (yr)				0.082
≤40	11 (5)	2 (20)	9 (4)	
>40	210 (95)	8 (80)	202 (96)	
Smoking				0.531^b^
Never (pack-year ≤5)	101 (46)	5 (50)	96 (46)	
Smoker (pack-year >5)	117 (53)	5 (50)	112 (53)	
unknown	3 (1)	5 (0)	3 (1)	
Serum CEA level				0.522
Normal	123 (56)	6 (60)	117 (55)	
Elevated	98 (44)	4 (40)	94 (45)	
Pathological stage				0.014
I	150 (68)	3 (30)	147 (70)	
II–IV	71 (32)	7 (70)	64 (30)	
Tumor size (mm)				0.034
≤30	159 (72)	10 (100)	149 (71)	
>30	62 (28)	0 (0)	62 (29)	
Pathological nodal status				0.002
N0	168 (76)	3 (30)	165 (78)	
N1/N2	53 (24)	7 (70)	46 (22)	
Lymphatic permeation				0.136
Positive	85 (38)	6 (60)	79 (37)	
Negative	136 (62)	4 (40)	132 (63)	
Vascular invasion				0.143
Positive	86 (39)	6 (60)	80 (38)	
Negative	135 (61)	4 (40)	131 (62)	

a Fisher’s exact test.

b *P* values were derived from a comparison between never smokers and smokers.

CEA, carcinoembryonic antigen.

**Table 4:** Characteristics of patients with *ALK* true and false positive tumors.

	Gender	Age (yr)	Smoking(Pack-Year)	Histology	Tumor size (mm)	pN status	p stage	Ly	V	ALK IHC (iScore)	ALK FISH	ALK RT-PCR
Test set												
P 1	F	49	10	Ad (solid with mucin)	30	N2	IV	+	+	3	+	E17ins38;A20^a^
P 2	F	64	0	Ad (AIS, nonmucinous)	5	N0	IA	−	−	3	+	V1
N 1	M	60	30	Squamous cell carcinoma.	50	N0	IB	+	+	1	−	−
N 2	M	69	100	Ad (solid = acinar>papillary)	55	N0	IIA	+	−	1	−	−
N 3	M	62	140	Small cell carcinoma	53	N2	IIIA	+	+	2	−	−
Validation set												
P 3	M	67	23	Ad (acinar>micropapillary)	21	N0	IA	−	+	3	+	V3a/b
P 4	M	34	13	Ad (papillary>micropapillary)	24	N2	IIIA	+	+	3	+	V3a/b
P 5	M	71	40	Ad (papillary>acinar>solid)	24	N2	IIIA	+	−	3	+	V1
P 6	F	75	0	Ad (papillary>acinar>lepidic)	13	N1	IIA	−	−	3	+	V2
P 7	F	41	0	Ad (acinar>papillary>lepidic>micropapillary)	22	N2	IIIA	+	+	3	+	V2
P 8	F	62	0	Ad (solid>acinar, cribriform)	12	N1	IIA	+	+	3	+	Not assessable
P 9	F	37	0	Ad (invasive mucinous)	16	N0	IA	−	−	3	+	Not assessable
P 10	M	63	30	Ad (solid>acinar)	15	N2	IIIA	+	+	3	+	Not assessable

a A novel *ALK* fusion variant.

pN status, pathological nodal status; Ly, lymphatic permeation; V, vascular invasion; IHC, immunohistochemistry;

FISH, fluorescent *in situ* hybridization; RT-PCR, reverse transcription-PCR; TP, true positive case; N, cases negative for *ALK* rearrangement;

Ad, adenocarcionoma; AIS, adenocarcinoma in situ.

## Discussion

An accurate, reliable, reproducible method for the detection of *ALK* rearrangement is essential for identifying NSCLC patients who are candidates for treatment with ALK inhibitor (crizotinib), a drug that has shown dramatic clinical response in a recent clinical trial [4]. Because the incidence of *ALK* rearrangement is low in unselected NSCLC patients (2–5%) [6]–[14], it is necessary to elucidate clinicopathological and molecular characteristics of ALK-positive lung cancer to improve screening efficiency. *ALK* rearrangement has been reported to be associated with younger patient age, never or light history of smoking, and adenocarcinoma histology [7]–[9], [12]–[14], [19]. Additionally, most ALK-positive lung cancer is mutually exclusive to *EGFR* and *KRAS* mutations [6], [7], [9], [12], [13], [19]. In the present study, the detection rate of *EML4-ALK* fusion gene increased from 1.0% (2/202 patients with unselected lung cancer) in the test set to 5.1% (8/158 patients with *EGFR* and *KRAS* mutation-negative adenocarcinoma) in the validation set. Our data suggested that considering histology and *EGFR* and *KRAS* mutation statuses enriches the ALK-positive population with minimal risk for an inappropriate exclusion of potentially positive patients.

In our series, there were no significant differences in age and smoking status between ALK-positive and ALK-negative patients. Therefore, we believe that clinical characteristics, such as age and smoking status, should not be used to select patients for ALK screening. Histologically, solid, acinar, cribriform growth pattern with or without signet ring cell features have been reported to be the morphological characteristics of ALK-positive lung cancers [7], [14], [19]. However, we found no specific morphological characteristic for ALK-positive adenocarcinomas, and most were histologically heterogeneous, i.e. a mixture of various growth patterns (Table 3). Therefore, morphological characteristics also should not be used for pre-selection.

Although FISH assay has been used for enrolling patients with ALK-positive tumors in clinical trials [4], a true gold standard method to determine *ALK* rearrangement has not been established. To date, there have only been a few reports examining *ALK* rearrangement in lung cancer simultaneously by IHC, FISH, and RT-PCR, thereby allowing a direct comparison of these assays [20]. In the present study, we simultaneously performed IHC, FISH, and RT-PCR for all patients of the test set, and performed both IHC and FISH for all patients of the validation set. The 10 positive and 350 negative results in FISH were completely matched with iScore 3 and 0, respectively. Therefore, confirmatory FISH could be skipped in cases with iScore 3 or 0, while it might be required in cases with iScore 2 or 1. If a case with non-adenocarcinoma is judged iScore 3, a confirmatory test should be done. Lung cancers without *ALK* rearrangement sometimes show positivity in highly-sensitive anti-ALK IHC, like iAEP, especially in cases with neuroendocrine differentiation (small-cell, large-cell neuroendocrine, and other carcinomas with focally neuroendocrine differentiation) [21].

In the present study, immunohistochemical identification of ALK-positive lung cancer was performed according to a scoring system. The system, iScore, was developed for anti-ALK immunohistochemistry with iAEP method. It was used for patient selection in the clinical trial for an ALK inhibitor (AF802/CH5424802) with an objective response rate 93.5%, indicating of the clinical usefulness of iAEP method (the scoring system used in the clinical trial was not yet called iScore at the time of the trial, but was the same in scoring criteria) [22]. Accordingly, the results of the IHC, FISH and RT-PCR assays were completely matched through various variants in the present study, while a study employing other settings in IHC and RT-PCR showed that the results of the 3 assays were concordant in variant 3 of EML4-ALK but not in variant 1 [20]. ALK-positivity rates in the present study, 2.8% in the whole population and 5.1% in the group of *EGFR* and *KRAS* mutation-negative adenocarcinomas are consistent with those obtained in the many previous studies. In terms of ALK-negative cases, all of the cases with iScore 0, 1 or 2 were found negative for *ALK* rearrangement, resulting in a 100% negative predictive value. Taken together, anti-ALK IHC with iAEP method judged by iScore is a clinically-validated reliable primary screening tool.

The iScore system may be similar to that for HER2 in breast cancer [23]. However, in contrast to the rate of equivocal results in HER2 IHC that require confirmatory FISH (∼10%), the rate of iScore 2 and 1 was only 0.83% (3 of 360) in the present study. In addition, in the cases with iScore 3, almost all tumor cells were immunostained, which is consistent with the view that all cancer cells of *ALK* rearranged cancers harbor *ALK* fusion genes, although the lower limit of positive tumor cells was set at 80% for iScore 3. Unlike other scoring systems for anti-ALK IHC, staining intensity, which may be a less objective indicator than positive tumor cell rate, is not basically considered in iScore, except for the cases showing “checker board pattern”. These features of iScore make it easy for investigators to score the specimens stained for ALK by iAEP method.

RT-PCR is theoretically the most reliable assay to detect mutant transcripts because it can demonstrate the direct evidence of the translocation [3], [15], [24]. However, in NSCLC, there are many variants of the *EML4*-*ALK* fusion, and *ALK* may sometimes have other fusion partners such as *TFG* [25], *KIF5B* [16], [26] and *KLC1* [27]. Therefore, RT-PCR may miss *ALK* fusions that are not specifically tested for. Therefore, of the 3 analyses, we believe RT-PCR should not be performed alone when a tissue is available for IHC or FISH.

Interestingly, although the size of *ALK*-positive adenocarcinoma was significantly smaller than *ALK*-negative adenocarcinoma, the proportion of lymph node involvement was significantly more frequent. This observation was consistent with a large-scale cohort study investigating the clinicopathological implication of *ALK* rearrangement in surgically resected lung cancer [28]. As Paik *et al.* described [28], *ALK*-positive adenocarcinomas may metastasized to lymph nodes early, despite the small size of the primary tumor.

Our diagnostic algorithm was proposed based on the data using surgically resected specimens. Therefore, it is absolutely useful to obtain the *ALK* status for further therapeutic planning in patients with postoperative recurrences. Sakairi *et al.* [29] reported that *EML4*-*ALK* fusion gene assessment by IHC, FISH, and RT-PCR was possible using small samples obtained by endobronchial ultrasound-guided transbronchial needle aspiration from lymph nodes with metastatic tumors. Therefore, our algorithm is highly likely to be applicable to patients with advanced or inoperable disease for whom only small samples (biopsy or cytology) are usually available.

**Figure 4:**
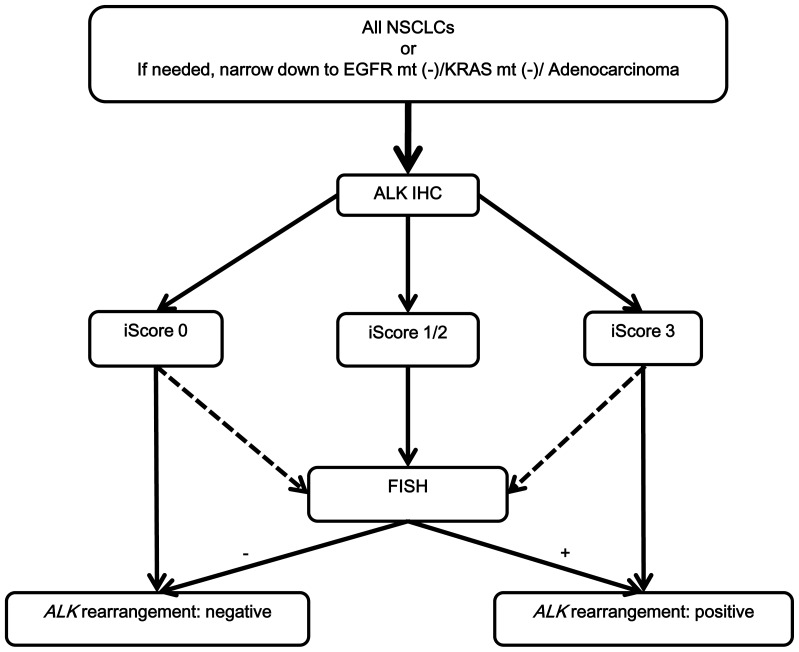
Diagnostic algorithm for the identification of *ALK* rearrangement in lung cancer. For cases with iScore 3 or 0, confirmatory FISH or RT-PCR can be skipped. However, if a case with non-adenocarcinoma is judged iScore 3, a confirmatory test should be done. Lung cancers without *ALK* rearrangement sometimes show positivity in highly-sensitive anti-ALK IHC, like iAEP, especially in cases with neuroendocrine differentiation (small-cell, large-cell neuroendocrine, and other carcinomas with focally neuroendocrine differentiation).

In summary, as far as anti-ALK immunohistochemistry is performed by a highly sensitive method like iAEP and the staining result is appropriately interpreted, screening for *ALK* rearrangement by IHC followed by confirmatory FISH is a reliable diagnostic algorithm (Figure 4). In addition, if it is needed, narrowing -down to patients with *EGFR* and *KRAS* mutation-negative adenocarcinomas seems rational, resulting in minimal risk for an inappropriate exclusion of potentially positive patients.
